# Late-Onset Puberty Induction by Transdermal Estrogen in Turner Syndrome Girls—A Longitudinal Study

**DOI:** 10.3389/fendo.2018.00023

**Published:** 2018-02-08

**Authors:** Aneta Monika Gawlik, Magdalena Hankus, Kamila Szeliga, Aleksandra Antosz, Tomasz Gawlik, Kamil Soltysik, Agnieszka Drosdzol-Cop, Krzysztof Wilk, Grzegorz Kudela, Tomasz Koszutski, Ewa Malecka-Tendera

**Affiliations:** ^1^Department of Pediatrics and Pediatric Endocrinology, School of Medicine in Katowice, Medical University of Silesia, Katowice, Poland; ^2^Nuclear Medicine and Endocrine Oncology Department, Maria Skłodowska-Curie Memorial Institute and Cancer Center, Gliwice Branch, Gliwice, Poland; ^3^Department of Anatomy and Molecular Cell Biology, Nagoya University Graduate School of Medicine, Nagoya, Japan; ^4^Chair of Woman's Health, Medical University of Silesia in Katowice, Katowice, Poland; ^5^Department of Obstetrics and Gynecology, The Boni Fratres Catoviensis, Katowice, Poland; ^6^Department of Pediatric Surgery and Urology, School of Medicine in Katowice, Medical University of Silesia, Katowice, Poland

**Keywords:** Turner syndrome, puberty induction, menarche, estrogen therapy, transdermal estrogen therapy, puberty, karyotype 45,X

## Abstract

**Objective:**

Estrogen replacement therapy (ERT) for Turner syndrome (TS) is a widely discussed topic; however, the optimal model of ERT for patients with delayed diagnosis and/or initiation of therapy is still unclear, mainly due to insufficient data. We present the results of a prospective observational single-center study in which the efficacy of late-onset puberty induction by one-regimen transdermal ERT in TS girls was evaluated.

**Methods:**

The analysis encompassed 49 TS girls (63.3% with 45,X) with hypergonadotropic hypogonadism in whom unified transdermal ERT protocol was used for puberty induction (first two months 12.5 μg/24 h, thereafter 25.0 μg/24 h until breakthrough bleeding). Clinical visits for examination and therapy modification took place every 3–6 months. Transabdominal pelvic ultrasound examinations were performed at least twice: at the beginning and at the end of follow-up.

**Results:**

The mean (SD) age at ERT induction was 15.1 (1.3) years. The duration of follow-up was 2.4 (1.1) years. Half of all the patients had at least B2 after 0.57 years, B3 after 1.1 years, B4 after 1.97 years, and menarche after 1.82 years from ERT initiation. With earlier initiation of ERT (≤14 years), B2 (*p* = 0.059) was achieved faster and B4 (*p* = 0.018) significantly slower than with the later start of ERT. Thirty-four (94.4%) patients had at least stage B3 at menarche. The karyotype, initial weight, and body mass index had no impact on puberty tempo during ERT. The uterine volume increased significantly during ERT in all the study group (*p* < 0.0001), and in half of the patients, the increase was at least 12.4-fold. It did not correlate with the duration of treatment (*p* = 0.84) or the dose of estradiol per kilogram (*p* = 0.78), nor did it depend on karyotype (*p* = 0.71) or age at ERT initiation (*p* = 0.28). There were no differences in ΔhSDS during ERT (*p* = 0.63) between the two age groups (ERT ≤14 and >14 years).

**Conclusion:**

The presented easy-to-use fixed-dose regimen for late-onset puberty induction allowed for a satisfactory rate of achieving subsequent puberty stages and did not influence the growth potential.

## Introduction

It is estimated that from 20 to almost 50%, Turner syndrome (TS) girls present some degree of pubertal development, with menarche in approximately 16–20%; however, this occurs nine times less frequently in girls with 45,X than in girls with a mosaic karyotype (1–3). Regular menstrual cycles are observed in 6% of the TS population (1) and only 2–5% achieve spontaneous pregnancy (4). Thus approximately 90% of TS girls and women require or will require estrogen replacement therapy (ERT) to initiate, progress, or maintain pubertal development. It is recommended that TS women should receive estrogen and progestin replacement, known to have long-term effects on puberty, fertility, bone health, metabolism, and psychological functioning (5, 6). The first-choice ERT regimen to initiate and progress pubertal development, and at the same time to mimic physiology and minimize risks, is still being discussed. The latest international guidelines recommend initiating ERT at the age of 12 years in the absence of spontaneous puberty and/or if the follicle-stimulating hormone levels are elevated (2, 7). In order to imitate natural development, an incremental increase in the dose is recommended over a period of 2–3 years until an adult dose has been reached. Although there is no evidence as to the superiority of any one ERT regimen, the transdermal route seems to be the most desirable (7).

The use of transdermal estradiol (E2) facilitates a more physiologic mode of delivery, without first-pass effects in the liver, avoiding unphysiological changes and hormone activity. Transdermal E2 results in faster bone accrual at the spine, increased uterine growth, and greater final height (8, 9). It also seems that transdermal E2 is safer in the context of thrombotic risk: thrombin generation is increased in postmenopausal women using oral estrogens. It could be mediated by the hepatic first-pass metabolism of estrone, the main metabolite of oral E2 (10).

In the absence of products designed specifically for puberty induction, the transdermal method offers the possibility to cut and modify the size of the patch in order to facilitate dose adjustment, although this is not recommended by the producers.

The form of ERT in girls with hypogonadism should mimic the physiology, preserve the growth potential, and, at the same time, minimize the risk of side effects. The 2017 guidelines recommend starting ERT at 11–12 years of age, with a dose increase every 6 months over a period of 2–3 years (7).

The most controversial, mainly due to lack of data, is the ERT model for TS girls with delayed diagnosis and/or the initiation of estrogen treatment. We present the results of a prospective observational single-center study in which the efficacy of late-onset puberty induction by one-regimen transdermal ERT in TS girls was evaluated.

## Patients and Methods

The study encompassed 62 consecutive TS girls who, between September 1997 and 2017, were treated with transdermal ERT at the Department of Pediatric Endocrinology in Katowice, Poland, using the same study protocol. The data of 13 patients were excluded from the analysis: eight girls were followed up for less than 1 year, three girls with spontaneous puberty presented premature ovarian failure symptoms, one girl initiated ERT in another clinical center, and one had incomplete clinical data. The final analysis encompassed 49 TS girls with hypergonadotropic hypogonadism in whom ERT was used for puberty induction. In all cases, TS was diagnosed based on a cytogenetic analysis using peripheral lymphocytes and was confirmed by karyotyping with routine G-banding according to the recommendations of the American College of Medical Genetics. In 31 girls (63.3%), 45,X karyotype was confirmed. Four girls with karyotype 45,X/46,XY had undergone gonadectomy due to the risk of malignant transformation (GK, TK). Forty-five (91.8%) had been treated with recombinant growth hormone (rGH). No data concerning the duration of rGH were available in four girls, and in one, the growth-promoting therapy was still ongoing.

Throughout the study, all the patients underwent two to four routine visits per year, during which a thorough clinical examination, including pubertal staging according to the method of Tanner (11) and anthropometric measurements, was performed by a single pediatric endocrinologist (AG).

Weight was measured with a precision to 100 g and height with Harpenden stadiometer to 0.1 cm. The body mass index (BMI) was calculated as weight (kg)/squared height (m). Height was expressed as standardized values (hSDS—height standard deviation score) based on the growth chart for healthy Polish girls (12). hSDS was calculated using the following formula: hSDS = child’s height − height for 50 pc/0.5 × (height 50 pc − height 3 pc).

In addition, the patients’ bone age (BA) was determined based on the X-ray of the non-dominating hand using the Greulich–Pyle Atlas (13).

Transabdominal pelvic ultrasound (US) examinations were performed at least twice, at the beginning and at the end of follow-up, using a 5-MHz convex transducer (Siemens Acuson Antares 5.0, Acuson Sequoia and Acuson 128 XP) (AD-C, KW). The uterine volume was determined using the formula *V* = *a* × *b* × *c* × 0.5 (*a*—diameter of longitudinal section, *b* and *c*—two diameters of transverse section) (14).

### ERT Regimen

Hypergonadotropic hypogonadism was diagnosed (2) in all our study patients. A unified protocol of transdermal ERT was applied: for the first 2 months, 12.5 µg of estradiol transdermally per 24 h (half of the patch releasing 25 µg or one-fourth of the patch releasing 50 µg), subsequently 25 µg of estradiol/24 h transdermally until breakthrough bleeding occurred, at which point the therapy was changed to cyclic estrogen–progesterone. The patch was replaced every 3.5 days (twice a week) (5). Compliance and side effects were assessed during every visit.

In order to assess the impact of karyotype and age at ERT initiation on the dynamics of puberty, the patients were arbitrarily divided into subsets with puberty induction of ≤14 or >14 years and with karyotype 45,X or non-45,X.

### Statistics and Data Analysis

Statistical analyses were performed with STATISTICA version 13.

Comparisons between two groups were performed with two-sided Student’s *t*-test or Fisher’s exact test, as appropriate. Kaplan–Meier analysis was applied to analyze the time course of breast development and menarche stimulation during treatment. Gehan’s Wilcoxon test was used to test the difference between groups in Kaplan–Meier analysis. Data are presented as means and SDs, medians, and ranges, and percentages, and unless stated otherwise, are presented in the text as mean (SD)/(range). *P*-values of <0.05 were considered to be significant.

The study was conducted in accordance with the Declaration of Helsinki and was approved by the Ethical Committee of Medical University of Silesia. Written informed consent was obtained from all patients aged over 16 and from their parents or legal custodians.

## Results

### Clinical Presentation

The mean (SD)/(range) age at TS diagnosis and/or of the first visit at the study center was 9.8 (4.5)/(0.4–17.6) years old. The age at the induction of transdermal ERT was 15.1 (1.3)/(11.7–17.8) years old. The duration of ERT follow-up was 2.4 (1.1)/(1.0–6.2) years. Before ERT induction, eight (16.3%) girls presented breast development at a stage higher than B1: B2 was observed in six girls, while B3 was observed in two girls. The duration of rGH therapy was 5.1 (2.8)/(1.1–10.9) years.

The clinical data of the 49 girls, also grouped by age and karyotype, are presented in Tables 1–3.

**Table 1:** Clinical data of the 49 Turner syndrome patients [45,X—31 (63.3%) and non-45,X—18 (36.7%)].

*n* = 49	Mean (SD)/(range)
Age at ERT start (years)	15.1 (1.3)/(11.7–17.8)
Age of the last visit (years)	17.5 (1.0)/(14.1–19.1)
Weight (kg)	46.7 (8.8)/(30–73.7)
BMI at ERT start (kg/m^2^)	20.6 (4.5)/(16.3–30)
BMI at the last visit (kg/m^2^)	26.8 (11.4)/(17.8–30.4)
hSDS at ERT start	−2.52 (1.16)/(−0.60 to −5.30)
hSDS at the last visit	−1.93 (0.99)/(0.23 to −5.23)
ΔhSDS	0.59 (0.67)/(−0.80–2.40)
BA at ERT start (years)	12.63 (0.92)/(10.0–14.0)
Uterus volume at ERT start (ml)	1.44 (1.87)/(0.12–8.8)
Uterus volume at the last visit (ml)	10.2 (7.3)/(1.7–40.4)
Increase of uterus volume during ERT (volume at the last visit/volume at ERT start)	19.2 (17.8)/(1.05–58.8)

*n, number of patients; ERT, estrogen replacement therapy; BMI, body mass index; hSDS, height standard deviation score; BA, bone age*.

### Dynamics of Breast Development during ERT

**Figure 1:**
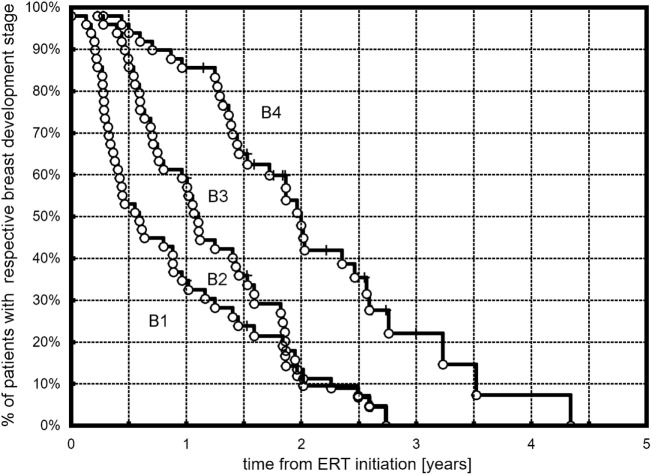
Kaplan–Meier plots showing the time course of B2, B3, and B4 development in the observed TS patients.

**Figure 2:**
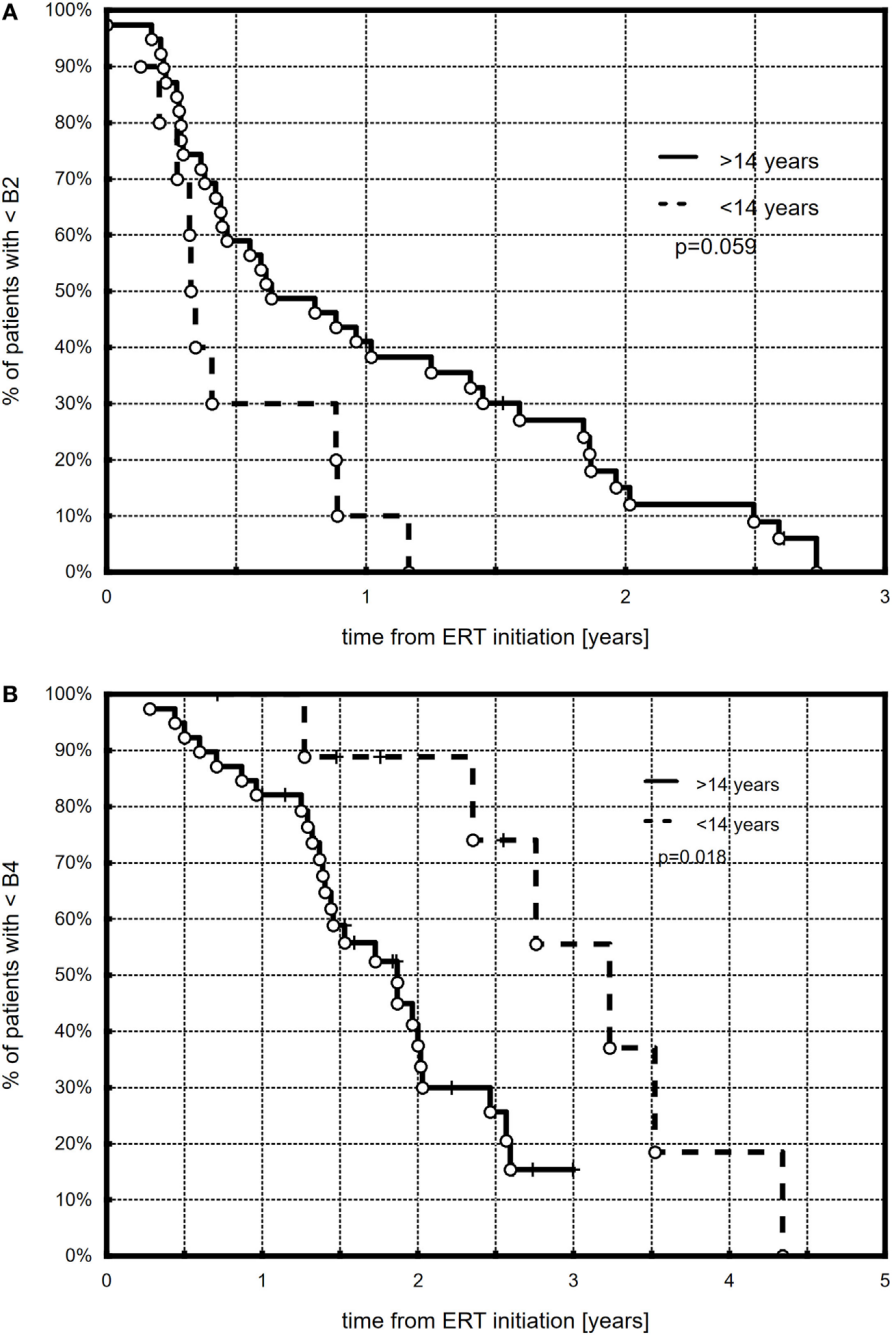
Kaplan–Meier plots showing the comparison between younger (≤14 years) and older (>14 years) patients with respect to B2 **(A)** and B4 **(B)** development.

All but three (6.1%) girls presented breast development with progression to at least B3. At the end of follow-up (during the last visit), stages 4 and 5 were observed, respectively, in 25 (52%) and 7 (14.3%) girls. The Kaplan–Meier curves showed that 50% of all the girls had at least B2 after 0.57 years, B3 after 1.1 years, and B4 after 1.97 years of ERT (Figure 1). A tendency in patients with earlier ERT initiation (≤14 years) to progress faster to B2 (Figure 2A, *p* = 0.059) and significantly slower to B4 (Figure 2B, *p* = 0.018) than in patients with late-onset ERT initiation (>14 years) was observed. The karyotype had no impact on the dynamics of achieving consecutive breast development stages during ERT.

**Figure 3:**
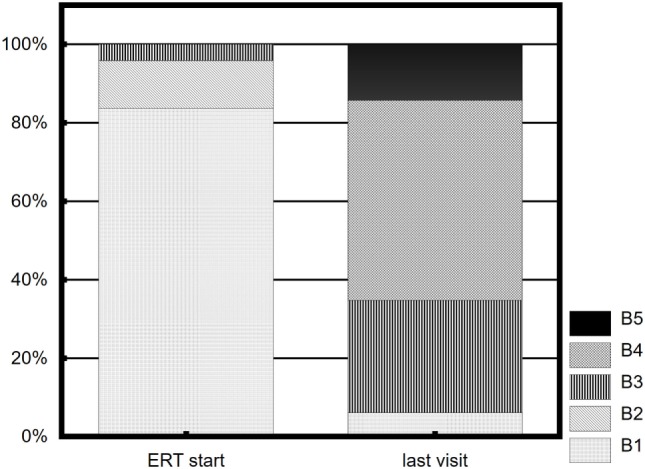
Distribution of breast development stages (B) at ERT initiation and at the last visit.

No breast tissue response to ERT was observed in three (6.1%) girls, with B1 at the last examination. All three girls belonged to the late-onset therapy group (>14 years), and two had karyotype 45,X. The distribution of B stages at ERT initiation and at the last visit is presented in Figure 3.

### Menarche during ERT

**Figure 4:**
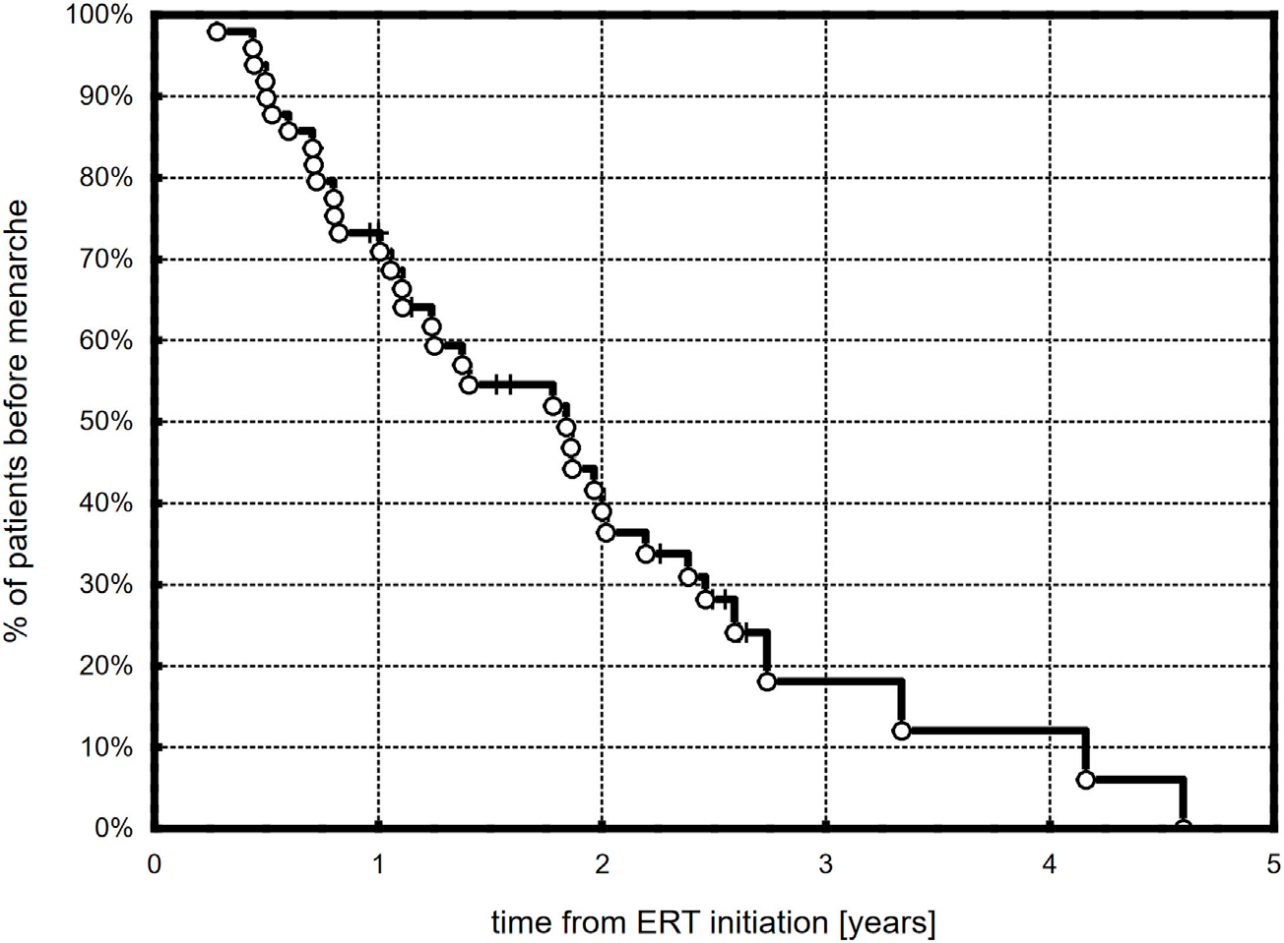
Kaplan–Meier plots showing the time course of menarche in the observed TS patients.

Menarche was observed in 36 (73.5%) girls during ERT, and it occurred after 1.5 (1.0)/(0.3–4.6) years. Based on the Kaplan–Meier curve, half of the patients were after their first menstruation at 1.82 years from the start of ERT (Figure 4). There were no differences in the time to menarche between girls with different karyotypes and with different age at therapy initiation. Most patients, 34 (94.4%), had at least stage B3 of breast development at menarche (B3, B4, and B5 in 17, 15, and 2 patients, respectively).

The girls’ initial weight or their BMI had no impact on the time of menarche.

### Dynamics of Uterine Development during ERT

The uterine volume at ERT initiation was comparable in the two age groups (ERT ≤14 and >14 years, *p* = 0.84) and was larger in non-45,X girls than in 45,X (*p* = 0.09). The initial uterine size did not correlate with the girls’ weight or hSDS (*p* = 0.78; *p* = 0.37). The uterine volume increased significantly during ERT in all the study group (*p* < 0.0001), in half at least 12.4-fold (Table 1). It did not correlate with the duration of treatment (*p* = 0.84) or the dose of estradiol per kilogram of the initial body weight (*p* = 0.78), and it was not dependent on karyotype (*p* = 0.71, Table 3) or age at ERT initiation (*p* = 0.28, Table 2).

**Table 2:** Clinical data of the 49 Turner syndrome patients grouped by age.

Karyotype 45,X; non-45,X *n* (%)	ERT start ≤14 years (*n* = 10) 8 (80%); 2 (20%)	ERT start >14 years (*n* = 39) 23 (59%); 6 (41%)	*p*-Value

	Mean (SD)/(range)	Mean (SD)/(range)	
Age at the first visit (years)	6.1 (4.4)/(04–13.6)	10.8 (4.1)/(1.1–17.6)	0.002
Age at ERT start (years)	13.3 (0.7)/(11.7–14.0)	15.5 (1.0)/(14.1–17.8)	0.000
Duration of follow-up (years)	3.4 (1.7)/(1.1–6.2)	2.2 (0.8)/(1.0–4.6)	0.048
Weight (kg)	45.1 (9.4)/(31.0–58.5)	47.2 (8.8)/(30.0–73.7)	NS
BMI at ERT start (kg/m^2^)	20.9 (3.4)/(16.3–25.9)	21.2 (3.0)/(17.1–30.0)	NS
BMI at the last visit (kg/m^2^)	21.5 (3.0)/(17.8–25.4)	23.0 (2.9)/(18.5–30.4)	NS
hSDS at ERT start	−2.36 (1.05)/(−4.58 to −1.00)	−2.56 (1.20)/(−5.30 to −0.60)	NS
hSDS at the last visit	−1.87 (0.98)/(−3.75 to −0.30)	−1.95 (1.00)/(−5.23–0.23)	NS
ΔhSDS	0.50 (0.77)/(−0.80–2.20)	0.61 (0.66)/(–0.75–2.40)	NS
BA at ERT start (years)	12.25 (0.82)/(11.0–14.0)	12.7 (0.9)/(10.0–14.0)	NS
Uterus volume at ERT start (ml)	1.54 (1.56)/(0.12–5.30)	1.41 (1.97)/(0.14–8.80)	NS
Uterus volume at the last visit (ml)	13.8 (14.3)/(1.7–40.4)	9.6 (5.1)/(2.2–22.1)	NS
Increase of uterus volume during ERT (volume at the last visit/volume at ERT start)	14.3 (9.8)/(1.1–31.1)	20.1 (19.0)/(1.05–58.8)	NS

*n, number of patients; ERT, estrogen replacement therapy; BMI, body mass index; hSDS, height standard deviation score; BA, bone age; NS, not significant*.

**Table 3:** Clinical data on the 49 Turner syndrome patients grouped by karyotype.

	45,X (*n* = 31)	Non-45,X (*n* = 18)	*p*-Value

	Mean (SD)/(range)	Mean (SD)/(range)	
Age at the first visit (years)	9.4 (5.0)/(0.4–16.7)	10.5 (3.6)/(4.6–17.6)	NS
Age at ERT start (years)	14.8 (1.3)/(11.7–17.3)	15.5 (1.2)/(13.0–17.8)	NS
Duration of follow-up (years)	2.7 (1.2)/(1.0–6.2)	2.0 (0.8)/(1.0–3.8)	0.038
Weight (kg)	47.5 (9.7)/(30.0–73.7)	45.5 (7.2)/(31.0–59.6)	NS
BMI at ERT start (kg/m^2^)	21.5 (3.5)/(16.3–30.0)	20.6 (2.3)/(17.4–25.4)	NS
BMI at the last visit (kg/m^2^)	22.8 (3.0)/(17.8–30.4)	22.5 (2.9)/(18.6–28.8)	NS
hSDS at ERT start	−2.46 (1.25)/(−5.30 to −0.60)	−2.63 (1.01)/(−4.58 to −1.08)	NS
hSDS at the last visit	−1.82 (1.00)/(0.23 to −5.23)	−2.12 (0.97)/(−4.00 to −0.50)	NS
ΔhSDS	0.64 (0.72)/(−0.80–2.40)	0.51 (0.59)/(−0.75–1.59)	NS
BA at ERT start (years)	12.52 (0.97)/(10.00–14.00)	12.83 (0.80)/(11.5–14.00)	NS
Uterus volume at ERT start (ml)	1.00 (1.17)/(0.12–6.32)	2.33 (2.67)/(0.14–8.80)	NS
Uterus volume at the last visit (ml)	10.4 (8.2)/(1.7–40.4)	10.0 (5.3)/(2.2–21.8)	NS
Increase of uterus volume during ERT (volume at the last visit/volume at ERT start)	18.4 (15.8)/(1–48.7)	20.8 (22.1)/(1.4–58.8)	NS

*n, number of patients; ERT, estrogen replacement therapy; BMI, body mass index; hSDS, height standard deviation score; BA, bone age; NS, not significant*.

### Height

There were no differences in ΔhSDS during ERT (*p* = 0.63, Table 2) between the two age groups (ERT ≤14 and >14 years).

### Side Effects of ERT

No estrogen-related adverse events were reported.

## Discussion

In this paper, we presented the results of a prospective observational study with 49 TS girls in whom the same model of ERT initiation was used. The mean age at pharmacological puberty initiation in our study group was over 15 years, and only one-fifth of the girls started ERT before the age of 14 years. In view of the recommendations, this is recognized as late-onset puberty induction. The use of a 2- to 3-year model for the induction of puberty in such conditions does not seem to be optimal, especially from the patient’s point of view.

Delayed ERT induction in our patients was mostly the result of late TS diagnosis and, consequently, of late onset of rGH therapy; in some cases, it was caused by the patient’s and/or her family’s reluctance for fear of adverse impact of estrogen on the final height. The age at the first visit in our center for girls with ERT initiation of >14 years old was significantly higher compared to that in girls with ERT before 14 years old. The karyotype, and thus, indirectly, the severity of phenotype presentation, had no impact. In most girls, the BA at the time of ERT initiation was assessed at 13 years of age.

Our observational study focused on the dynamics of puberty advancement during ERT. The applied protocol of transdermal therapy was the authors’ original concept (AG) and was not modeled on any previous studies. Our center was one of the first to start transdermal estradiol therapy in TS girls, almost 20 years ago. We found that half of all the treated girls had at least Tanner stages B2, B3, or B4 after 0.57, 1.1, and 1.97 years of treatment. Girls with ERT initiation of ≤14 years tended to achieve B2 faster and B4 significantly slower than girls with late-onset ERT initiation. At the end of the follow-up, stages 4 and 5 of breast development were observed, respectively, in 52 and 14.3% of the girls. Menarche occurred in more than 70% of the girls. Half of the girls were after their first menstruation at 1.82 years from the start of ERT.

A review of the literature showed studies presenting different schemes of ERT for puberty induction, some related to puberty progression (8, 15–20). Each of these regimens was different and difficult to compare, also due to the different age at ERT initiation. In the study by Nabhan et al., transdermal estradiol therapy in 14-year-old patients was compared to conjugated estrogen. It was characterized by a quite high and fast increase in dose, even in comparison to our protocol. By using this 1-year protocol (first 6 months, 25 μg/day transdermally, thereafter next 6 months, 37.5 μg/day), the breast stage increased more progressively than in our study, and after 1 year, TS patients were of Tanner stage 3 or 4. Breakthrough bleeding occurred in four of their six girls, and it took place earlier than in our study (8).

Other studies were even less comparable with regard to the study protocol and the age at pharmacological puberty induction. Bannink et al. used increasing doses of oral estradiol at the mean age of 12.7 years and observed breast development comparable to normal with a 2-year delay (17). A Dutch study showed that treatment with micronized E2 started at a mean age of 12.7 years facilitated reaching B2 just before 13 years and B4 at a mean age of 14.8 years (16). A 2-year treatment with oral E2 (47 girls, age 13–14), monitored by a Spanish Turner working group showed that using either a fixed or an individualized dose allowed to attain B4 or B5 in 2 years. In the fixed-dose model, a shorter time was needed (2.0 vs. 2.2 years), and a tendency to a higher proportion of girls with a minimum B4 at the study end was observed (65 vs. 42%) (19). Piippo et al. used percutaneous E2 gel for puberty induction in 23 girls of a median age of 13.6 years, with the development of secondary sexual characteristics and uterine growth proceeding gradually, mimicking natural puberty. At the end of the 5-year treatment, all girls reached at least B4. In three patients, spontaneous bleeding occurred after 6 months, and in one after 1.25 years (20).

Despite the different ERT regimens used in the cited studies, the dynamics of breast development was similar to our study: stage B2 during the first months and B4 after approximately 2 years. This is comparable to spontaneous puberty. In some girls, resistance to estradiol therapy was noticed (8). In our study, this occurred in three girls, interestingly, all with late onset of therapy induction.

A unique schedule of ERT was presented by a Swedish group. In the study by Ankarberg-Lindgren et al., with the nocturnal application of transdermal estradiol (0.08–0.12 µg/kg), stage B2 occurred at 3–6 months from the first-night administration of patches in most of the 15 patients with hypogonadism; B3 was observed in seven girls after 3.5–29 months. However, similar to our observation, the authors did not find a correlation between the given dose per kilogram and the rate of progression of breast development (18).

One of the most important issues in the context of ERT is uterine development, both as a marker of therapy effectiveness and as a chance for future *in vitro* fertilization procedures. According to Bakalov and McDonnell, TS women may develop a normal uterus even at a late start of HRT, given the adequate duration of treatment and regardless of karyotype (21, 22). This is in contrast with other studies. Doerr et al. found that only TS women with karyotype 45,X/46,XX had a normal uterine size, whereas approximately 18 and 25% of TS women with karyotype 45,X had a uterine volume and length below −2 SD (23). In a Danish study, the mean uterine volumes by MRI and US in fully matured TS girls were lower than in controls despite appropriate hormonal therapy in TS (24). Transdermal ERT seems to be more effective in uterine size increase compared to conjugated estrogens (8). By using estradiol gel, the uterine volume increased from 5.5 to 31.5 ml with a range of 8.2–82.8 ml (20). Our results were in line with the literature. The initial uterine size did not depend on the girls’ weight or height, and girls with 45,X tended to present smaller uterine dimensions. Using fixed transdermal therapy, we observed a marked increase in the uterine volume: at least 12.4-fold in half of our patients. Interestingly, the increase did not correlate with the duration of treatment or the dose of estradiol per kilogram of the initial body weight. Moreover, it did not depend on the age at ERT initiation. Similar to previously published results, the increase in uterine size did not depend on the karyotype (8, 25).

Ideally, ERT should mimic physiology, facilitating normal-pace puberty and promoting growth. This is possible if TS is diagnosed early. However, there are no data to support the specifics of ERT timing and doses in cases of delayed diagnosis and puberty induction. The decision is individual and based on the doctor’s experience. In this paper, we presented a model for late-onset puberty induction which resulted in a satisfactory rate of achieving subsequent puberty stages and which did not influence the growth potential. What is important, in the context of compliance, this regimen was easy to use and was well tolerated by the patients.

## Author Contributions

AG and MH designed the study, analyzed the database, and wrote the manuscript. KS, TG and KS prepared and analyzed the patients’ database and wrote the manuscript. AD-C and KW participated as gynecology consultants. EM-T, AA, GK, and TK collaborated in designing the work and drafting the manuscript.

## Conflict of Interest Statement

The authors declare that there is no conflict of interest that could be perceived as prejudicing the impartiality of the study.
